# Model Lipid Raft Membranes for Embedding Integral Membrane Proteins: Reconstitution of HMG-CoA Reductase and Its Inhibition by Statins

**DOI:** 10.1021/acs.langmuir.2c02115

**Published:** 2022-11-06

**Authors:** Michalina Zaborowska, Dorota Matyszewska, Renata Bilewicz

**Affiliations:** † Faculty of Chemistry, University of Warsaw, Pasteura 1, 02093Warsaw, Poland; ‡ Faculty of Chemistry, Biological and Chemical Research Centre, University of Warsaw, Żwirki i Wigury 101, 02089Warsaw, Poland

## Abstract

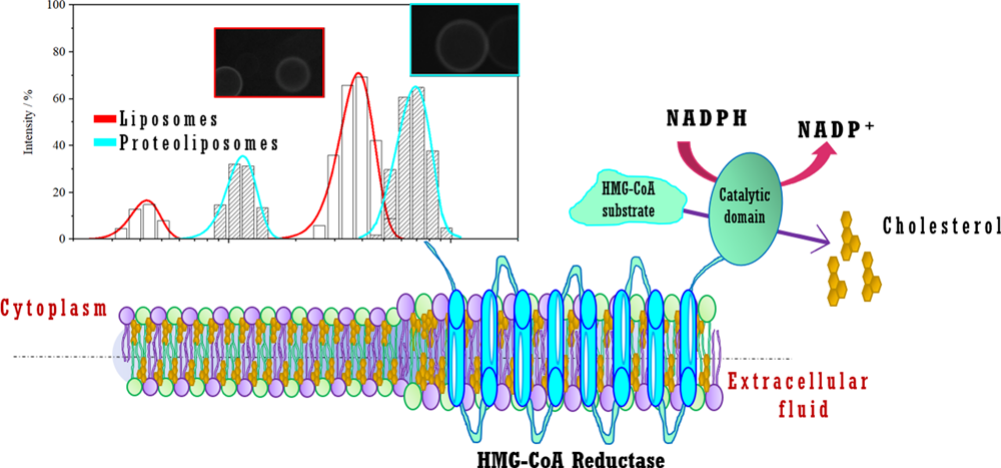

For the first time, HMG-CoA reductase, the membrane protein responsible for cholesterol synthesis, was incorporated into a lipid membrane consisting of DOPC:Chol:SM at a 1:1:1 molar ratio, which mimics the lipid rafts of cell membranes. The membrane containing the protein was generated in the form of either a proteoliposomes or a film obtained by spreading the proteoliposomes at the air–water interface to prepare a protein-rich and stable lipid layer over time. The lipid vesicle parameters were characterized using dynamic light scattering (DLS) and fluorescence microscopy. The incorporation of HMG-CoA reductase was reflected in the increased size of the proteoliposomes compared to that of the empty liposomes of model rafts. Enzyme reconstitution was confirmed by measuring the activity of NADPH, which participates in the catalytic process. The thin lipid raft films formed by spreading liposomes and proteoliposomes at the air–water interface were investigated using the Langmuir technique. The activities of the HMG-CoA reductase films were preserved over time, and the two lipid raft systems, nanoparticles and films, were exposed to solutions of fluvastatin, a HMG-CoA reductase inhibitor commonly used in the treatment of hypercholesterolemia. Both lipid raft systems constructed were useful membrane models for the determination of reductase activity and for monitoring the statin inhibitory effects and may be used for investigating other integral membrane proteins during exposure to inhibitors/activators considered to be potential drugs.

## Introduction

Insertion into lipid membranes is necessary for most membrane proteins to fully express their properties and activities. The successful reconstitution of proteins in model membranes depends on their physicochemical properties and the choice of membrane components. Studies of membrane proteins in a suitable lipid environment would allow us to describe the mechanisms underlying lipid–protein interactions and understand the influence of other molecules (e.g., drugs) on protein activity.^1^ If the protein exhibits hydrophobic properties, as in the case of gramicidin^2−4^ or the channel proteins described by Walewska et al.,^5^ then it may be reconstituted directly in the lipid mixture. In such cases, it is denatured in an organic solvent and then spontaneously incorporated into predeposited lipid layers, as described by Puiggalí-Jou.^6,7^ Outer membrane proteins have been incorporated using this method. Other approaches include the incorporation of proteins into bicelles,^8^ lipid or polymer nanodisks,^9^ or nanocarriers, such as cubic-phase nanoparticles.^5,10^ A common method involves the reconstitution of proteins into liposomes to obtain proteoliposomes.^1,11−13^ The incorporation of proteins into the structure of liposomes can be performed through direct and indirect reconstitution using a detergent (e.g., Triton X-100 or C12E8).^12^ Indirect reconstitution involves the formation of liposomes by the hydration of a thin lipid film with a solution in the presence of a detergent. Such a method allows for the expansion of the lipid structures so that proteins can more easily penetrate the lipid layer. The disadvantage of this method is the need to remove the detergent from the adsorbent, which can also remove or denature proteins. Where possible, it is better to use the protein reconstitution method without the use of detergents, which significantly reduces the interference with the lipid–protein system. Liposomes prepared using the Bangham method^14,15^ (without using detergents) are a promising biomimetic model for protein reconstitution. The advantage of this approach is the possibility of spreading them to form planar lipid layers, both on a solid substrate and at the air–solution interface.^16^ Another method of embedding the protein in model biological membranes at the air–water interface is the spontaneous incorporation of the protein into a lipid monolayer from the subphase, as described by Caseli et al.^17,18^ The gold standard for the reconstitution of ion-channel proteins is their incorporation into planar free-standing lipid bilayers (PLBs), also called black lipid membranes (BLMs).^19−21^ The PLB technique allows for easy access to both sides of the reconstituted channel and the application of electrophysiological techniques to characterize the activities of single channels, such as mitochondrial potassium channels.^5,22^ This technique not only helped to determine the biophysical properties of the channels, such as the conductance and selectivity, but was also crucial for describing the mitochondrial potassium channel pharmacology.

**Figure 1:**
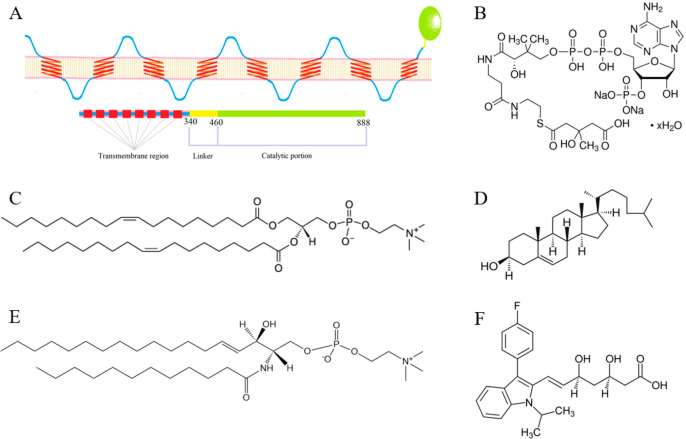
(A) HMG-CoA reductase anchored in the endoplasmic reticulum (ER) membrane. (B) Chemical structure of dl-3-hydroxy-3-methylglutaryl coenzyme A and the chemical structures of lipids: (C) 1,2-dioleoyl-*sn*-glycero-3-phosphocholine (DOPC), (D) cholesterol (Chol), (E) sphingomyelin (SM), and the statin (F) fluvastatin.

3-Hydroxy-3-methylglutaryl coenzyme A reductase (HMG-CoA reductase) is a transmembrane glycoprotein located on the membranes of the endoplasmic reticulum (ER) (Figure 1A) and is responsible for the biosynthesis of cholesterol in liver cells (hepatocytes).^23,24^ Initially, it was assumed that this protein has seven transmembrane spans;^25^ however, the presence of eight transmembrane spans has been postulated.^26^ This protein has two main domains: the N-terminal (amino acids 1–339) is the sterol-sensing domain, and the C-terminal (amino acids 460–888) is the catalytic portion.

Cardiovascular diseases (CVD) continue to be the leading cause of mortality worldwide.^27^ They are mainly caused by hypercholesterolemia,^28,29^ which is an elevated level of cholesterol in the blood plasma. Research on hypercholesterolemia has focused mainly on lowering the LDL cholesterol levels. Statins inhibit the catalytic properties of HMG-CoA reductase due to their affinity for the active site of the protein, which is larger than the substrate involved in cholesterol biosynthesis.^30−33^

Due to the complex nature of transmembrane HMG-CoA reductase, the proteoliposome approach was chosen to reconstitute this enzyme. We have recently demonstrated the successful use of this method for Na^+^/K^+^-ATPase protein incorporation.^13^ We have also shown the possibility of spreading proteoliposomes at the air–water interface using the Langmuir technique. This approach allowed us to investigate the lipid–protein interactions in thin films without any solid support and to demonstrate that the protein activity at the air–water interface can be retained. In this work, we show that the previously described method can be successfully applied to other proteins, such as HMG-CoA reductase. Moreover, we show that spreading proteoliposomes at the air–water interface allows not only for the preservation of protein activity but also for the study of the inhibition of this protein by statins. To mimic real membrane conditions as much as possible, HMG-CoA reductase was first reconstituted into liposomes composed of DOPC:Chol:SM in a molar ratio of 1:1:1 (Figure 1). In our recent studies, we showed that this lipid ratio generates the biochemical characteristics of the natural lipid raft microdomains.^34−36^ We have also demonstrated that the model lipid rafts themselves are significantly affected by the presence of statins.^36,37^ Since raft microdomains are anchor sites for HMG-CoA reductase, the aim of the present study was to construct a model system containing lipid rafts with embedded HMG-CoA reductase protein to study the effect of statins on the lipid rafts of cell membranes *in vitro*. We showed that the protein remains active in such a model system, even after being spread at the air–water interface. The constructed model membrane is a convenient tool for studying statin-induced HMG-CoA reductase inhibition.

## Experimental Methods

### Materials

Lipids used for the preparation of liposomes (1,2-dioleoyl-*sn*-glycero-3-phosphocholine (DOPC) and sphingomyelin (SM), purity ≥99%) were purchased from Avanti Polar Lipids (USA), and cholesterol was purchased from Sigma-Aldrich (purity ≥99%). All solvents were anhydrous and of high purity (HPLC-grade) and were obtained from Sigma-Aldrich. Lipid solutions at concentrations of 1 and 5 mg/mL were prepared by dissolving the appropriate amount of lipid in chloroform (DOPC, Chol) and in a 4:1 mixture of chloroform/methanol (SM). Appropriate amounts of the solutions were used to prepare the 1:1:1 DOPC:Chol:SM lipid mixture (characteristic of lipid rafts) so that the total lipid concentration in the sample was approximately 5 mg/mL. 3-Hydroxy-3-methylglutaryl coenzyme A reductase (HMG-CoA reductase, 76 kDa) in the form of a protein solution in Tris buffer (50 mM) supplemented with DTT, a 1:200 protease inhibitor cocktail, and glycerol were purchased from Sigma-Aldrich. Tris-HCl buffer (pH 7.4) in Milli-Q water (resistivity 18.2 MΩ, Millipore, Bedford, MA, USA) was used as the subphase (50 mM + 130 mM NaCl) in Langmuir experiments and as a solution for the hydration of the dry lipid film during the preparation of liposomes (20 mM, without NaCl). The available assay kit for activity measurements (Sigma-Aldrich) includes the following: an assay buffer, NADPH, the substrate solution (HMG-CoA), the catalytic domain of HMG-CoA reductase (0.50–0.70 mg/mL), and the statin. NADPH and dl-3-hydroxy-3-methylglutaryl coenzyme A sodium salt hydrate (HMG-CoA substrate) were obtained from Sigma-Aldrich for the activity measurements. Fluvastatin, which was used in the inhibition experiments at a concentration of 10^–5^ M, which corresponds to the concentration most often used in model membranes and *in vitro* studies,^37,38^ was obtained from Sigma-Aldrich.

### Methods

#### Preparation of Liposomes and Proteoliposomes

The Bangham method was used to obtain liposomes. The lipid mixture (DOPC/Chol/SM at 1:1:1, *C* = 5 mg/mL) was placed in Eppendorf vials, and the organic solvent was evaporated under a stream of argon with limited air access to avoid oxidizing the double bond in the DOPC molecules.^39,40^ The sample was then placed in a desiccator for approximately 3 h to remove the organic residues. The hydration of the thin lipid film involves adding an appropriate amount of Tris-HCl buffer solution (20 mM, pH 7.4) to the sample. The prepared sample was then placed in an ultrasonic bath for approximately 1.5 h at 37 °C (above the phase-transition temperature for the employed lipids).^41,42^ For the preparation of the proteoliposomes, HMG-CoA reductase was added to a previously prepared liposome sample (0.02 mol % vs lipids) and the solution was then shaken for approximately 2 h (direct method). An attempt was made to perform indirect reconstitution using the C12E8 detergent. However, the process of detergent removal using biobeads resulted in the removal of proteins from the lipid structure.

#### Dynamic Light Scattering (DLS) Measurements

DLS measurements were performed using a Zetasizer Nano ZSP (Malvern Panalytical, Malvern, U.K.) at 25 °C. The sizes of the liposomes and proteoliposomes were measured in a Tris-HCl buffer solution (50 mM, pH 7.4) in quartz cuvettes. The size distribution of the liposomes and proteoliposomes was expressed as a hydrodynamic diameter distribution.^43^

#### Fluorescence Microscopy

The liposomes and proteoliposomes were doped with 1 mol % 25-[*N*-[(7-nitro-2-1,3-benzoxadiazol-4-yl)methyl]amino]-27-norcholesterol (NBD-Chol). A Millicell EZ SLIDE eight-well glass (Merck KGaA, Darmstadt, Germany) was used to observe the vesicles. To avoid disturbing the properties of the HMG-CoA reductase transmembrane protein, we did not use an electroformation procedure to obtain GUVs.^44^ Instead, 50 μL of the liposome/proteoliposome suspension was placed in a well containing 200 μL of Tris-HCl buffer solution (50 mM, pH 7.4). Fluorescent images were captured using the JENOPTIK GRYPHAX software. The vesicles were monitored in real time for 15 min using an inverted confocal microscope (Nikon Eclipse Ni-U, Tokyo, Japan). Experiments were performed at room temperature (21 ± 1 °C).

#### Langmuir Monolayers

The surface pressure–area of the trough (*π–A*) isotherms were recorded using a KSV Nima (Finland) Langmuir trough with a total area of 243 cm^2^. The setup consisted of two hydrophilic barriers, a Wilhelmy microbalance with a Wilhelmy plate, and a computer with the KSV software. The subphase used in the experiments was a 50 mM Tris-HCl buffer with 130 mM NaCl (pH 7.4). NaCl was added to the subphase to increase the ionic strength and promote the spreading of lipid vesicles at the air–water interface.^16^ The solution containing the lipid mixture, liposomes, and proteoliposomes was applied to the subphase using a Hamilton microsyringe. Depending on the lipid concentration, an appropriate amount of the 1:1:1 DOPC/Chol/SM regular lipidic solution (in an organic solvent, chloroform or chloroform/methanol (*C* = 1 mg/mL)) was used, and the experiment was conducted as described previously.^37,45^ However, it is difficult to estimate the exact lipid concentration in vesicles (liposomes and proteoliposomes) because of the complex preparation protocol. Therefore, an exact amount of 200 μL of the liposome/proteoliposome solution was applied each time. A waiting time of 10 min was sufficient for the vesicle fusion process. Next, the dependence of the surface pressure on the trough area (cm^2^) was recorded. Each experiment was repeated at least 15 times to ensure reproducibility. All of the measurements were performed at room temperature (21 ± 1 °C).

An important parameter characterizing the layers at the air–water interface is the compression modulus (*C*_s_^–1^), which describes the elastic properties of the layers.^46^ It was calculated according to the formula below, where *A* is the area per molecule and π is the surface pressure.
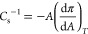
1Depending on the maximum value of this parameter (*C*_s_^–1^_max_), the obtained layer was assigned to a given phase: the gas phase (0–12.5 mN/m, G), liquid-expanded phase (12.5–100 mN/m, LE), liquid-condensed phase (100–250 mN/m, LC), or solid phase (>250 mN/m, S).^47^

#### π–*t* Measurements

Another type of measurement consists of the surface pressure changes over time. These measurements showed the stability of the layers obtained by the spreading of liposomes and proteoliposomes at the air–water interface. The measurements were performed by spreading the appropriate amount of liposomes/proteoliposomes at the air–water interface and by compressing the layers first to a surface pressure of 30 mN/m. This surface pressure value corresponds to the physiological conditions in the body, according to the *in vivo* elastic properties and organization of lipids.^48,49^ From that moment, the barriers remained still, and the changes in the surface pressure were observed over time.

#### Brewster Angle Microscopy

The Brewster angle microscopy (BAM) images were obtained using the Nanofilm Ep3 setup with an UltraBAM objective (Accurion, Germany). The actual size of the images is 800 μm × 430 μm (with the lateral resolution of 2 μm). BAM images were recorded simultaneously with the compression of the layers on the Langmuir trough.

#### Measurements of the Activity and Inhibition of HMG-CoA Reductase: UV–Vis Spectrophotometry

Activity measurements were based on a four-electron reaction catalyzed by HMG-CoA reductase. The substrate used for this reaction was HMG-CoA (Figure 1B). In the presence of a biocatalyst (HMG-CoA reductase), NADPH is oxidized to NADP^+^, while the other reaction products are mevalonate and CoA-SH.^24^
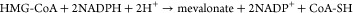
2The maximum absorbance was measured at 340 nm, which is correlated with the NADPH concentration. When the correct NADPH oxidation reaction occurred, a decrease in the absorbance value was observed, as expected in the presence of HMG-CoA reductase. The measured samples included: (1) the HMG-CoA reductase solution, (2) the proteoliposome suspension (with HMG-CoA reductase reconstituted into liposomes), and (3) the samples taken from the subphase after proteoliposome spreading at the air–solution interface. The concentration of NADPH in the UV 96-well microplates was 0.4 mM for each experiment. Measurements were performed using a Synergy LUX Multimode reader (BioTek Inc., USA) spectrophotometer.

We first measured the HMG-CoA reductase activity using the assay kit procedure, except that instead of the HMG-CoA reductase catalytic domain provided in the assay kit, a protein solution containing the whole structure (3-hydroxy-3-methylglutaryl coenzyme A reductase, HMG-CoA reductase), which was obtained from Sigma-Aldrich, was used. The same protein solution was also employed for the preparation of proteoliposomes; therefore, it was important to measure its activity at the very beginning. According to the protocol (HMG-CoA reductase assay kit (cat. no. CS1090)), which we strictly followed, the appropriate amount of Tris-HCl buffer (50 mM, without NaCl), NADPH (20 mM), 12 μL of HMG-CoA substrate, and the appropriate amount of HMG-CoA reductase protein were added to a 96-well microplate. Based on preliminary tests, it was estimated that the activity measured in 5 μL of the HMG-CoA reductase solution (0.50–0.70 mg/mL) corresponded to the same activity measured in 2 μL of the catalytic domain solution (0.50–0.70 mg/mL) provided in the assay kit. The time range was 2–30 min. The lower limit considers the sample preparation time and the mixing time recommended by the manufacturer. UV–Vis measurements were performed over 30 min to examine the changes in protein activity over time. A protocol similar to the one described above was used to measure the activity of the proteoliposome suspension. For this purpose, 60 μL of the proteoliposome solution was added to the measuring cell, and the amount of protein in the measuring cell was estimated to be the same as in the previous case. The third type of activity measurement was performed after spreading the proteoliposomes at the air–water interface. A mixture of the following components was used as the subphase on which the proteoliposomes were spread: NADPH (0.4 mM), HMG-CoA substrate (0.4 mM), and Tris-HCl buffer (50 mM + 130 mM NaCl, pH 7.4). After spreading 200 μL of the proteoliposome solution, the experiment was carried out in the same manner as that described above for the π–*t* measurements. The layer was compressed to a surface pressure of 30 mN/m. After monitoring the changes in surface pressure for 10 min, 200 μL of the subphase underneath the monolayer was taken using a syringe for the activity spectrophotometric measurement.

The enzyme activity was determined using the equation provided by the manufacturer (Sigma-Aldrich)^50^
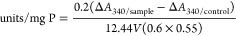
3where *ΔA*_340_ is the absolute difference between the initial absorbance and the absorbance at the subsequent time (10 min). Here, sample refers to the presence of HMG-CoA reductase and control refers to the absence of a reaction. The extinction coefficient for NADPH at 340 nm is 6.22 mM^–1^ cm^–1^ (since two moles of NADPH are consumed in the reaction, its value is 12.44); 0.2 mL is the total volume of the reaction; *V* is the volume of the enzyme used in the assay (mL); 0.6 is the enzyme concentration in mg of protein (mg P)/mL; and 0.55 cm is the value of the light path for the plates. The results are presented as μmol/min/mg protein (units/mg P).

Inhibition measurements were performed by adding appropriate volumes of fluvastatin to the measuring solutions to a final concentration of 10^–5^ M. The results of the inhibition of fluvastatin (sample) are presented in terms of % inhibition compared to that of the control sample, when the NADPH oxidation reaction did not occur (control). For this purpose, the following equation was used:^51,52^
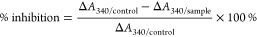
4All activity measurements for each system were repeated at least three times. The average and standard deviation were then calculated.

## Results and Discussion

### Liposome and Proteoliposome Preparation and Characterization

**Figure 2:**
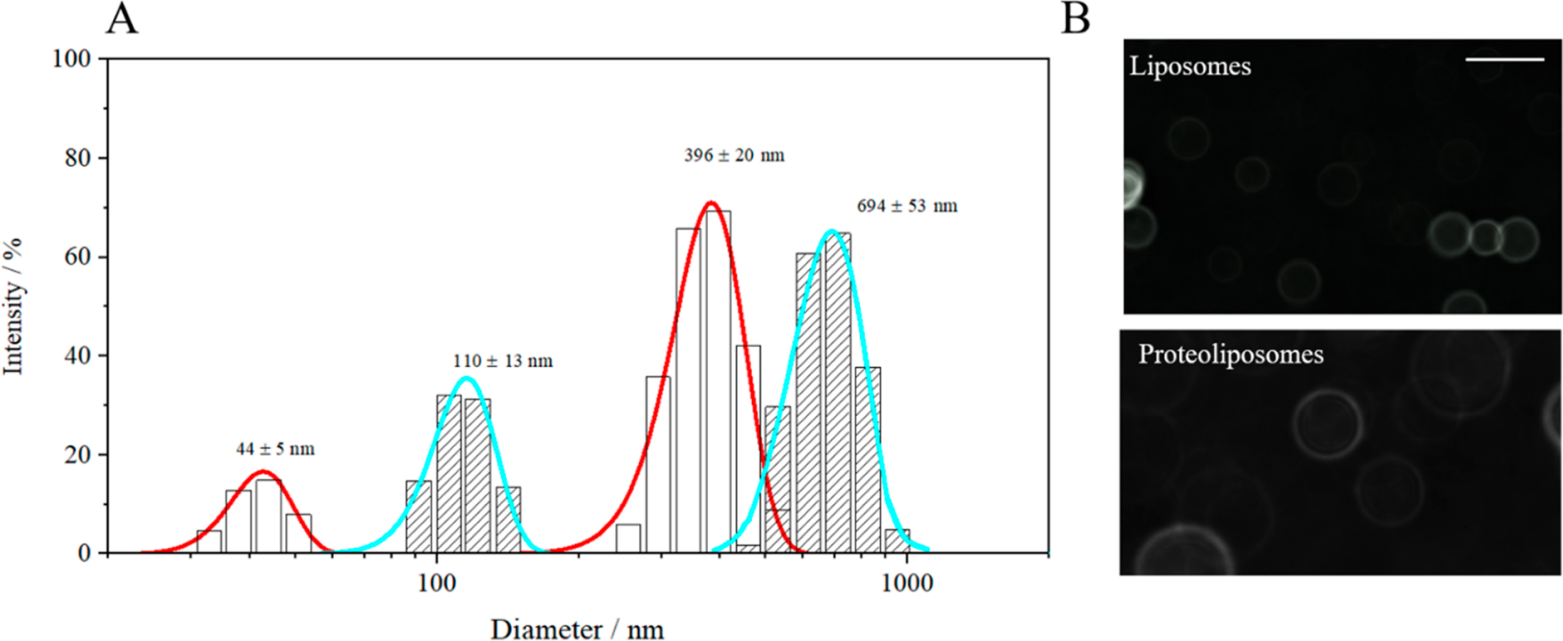
(A) Calculated Gaussian distributions of the particle sizes, as determined using DLS for the 1:1:1 DOPC/Chol/SM liposomes (red) and proteoliposomes with HMG-CoA reductase (0.02 mol %) (blue). (B) Visualization of the liposomes and proteoliposomes doped with NBD-cholesterol using fluorescence microscopy. The scale bar corresponds to 500 nm.

The sizes of the liposomes and proteoliposomes were evaluated using DLS (Figure 2). The average diameter of the liposomes was approximately 396 nm, with some smaller structures of 44 nm.

Such a size distribution is often connected with the formation of small unilamellar vesicles (SUVs) during the formation of large unilamellar vesicles (LUVs), when the vesicles are formed during sonication.^53,54^ The possibility of not all lipids binding to liposomes and the presence of smaller liposome clusters cannot be excluded. The presence of structures larger than those usually observed (of approximately 200 nm)^55^ may be related to the repulsive forces between the lipid components and/or the presence of double bonds in the DOPC lipid molecules, as discussed elsewhere.^6,36,51^ The direct reconstitution of HMG-CoA reductase (i.e., without using a detergent) results in structures with a maximum diameter of 694 nm, which means that the vesicles are almost twice as large as those obtained through indirect reconstitution. Moreover, HMG-CoA reductase has a high affinity for the lipid membrane. The bimodal size distribution observed here, with two clearly distinguished peaks,^56−58^ is representative of well-formed particles with different hydrodynamic diameters.^59^ This phenomenon can be explained by the formation of proteoliposomes with the desired composition and, simultaneously, by the limited transport between protein molecules and liposomes. The limited growth of the particles may also result from the low solubility of the lipid structures in the aqueous phase. A similar behavior was noted for the incorporation of cholesterol oxidase into the lipid matrix,^56^ which can be additionally explained by the formation of protein aggregates at the surface of the lipid vesicles. The bimodal particle size distribution depends on the lipid–protein ratio and occurs when there are significantly more lipids than proteins.^59^ This also depends on the sonication time.^60^

Fluorescence microscopy was employed to confirm the correct shape of the liposomes (Figure 2B). Both liposomes and proteoliposomes are characterized by a high curvature regularity, and their sizes correspond to those determined using the DLS method. The presence of HMG-CoA reductase did not significantly affect the phase contrast of the spherical structures obtained. The fluorescence microscopy images presented in the figure above reflect the variety of the liposomes and proteoliposomes. Figure S1 presents the stability of the structures in time. These images highlight the fact that the vesicles do not change their phase contrast or shape. Additionally, the vesicular structures of the proteoliposomes were visualized using cryo-TEM (Figure S2A). Both methods of lipid vesicles visualization convince us that no aggregates were formed.

### Characteristics of the Liposomes and Proteoliposomes Spread at the Air–Water Interface

**Figure 3:**
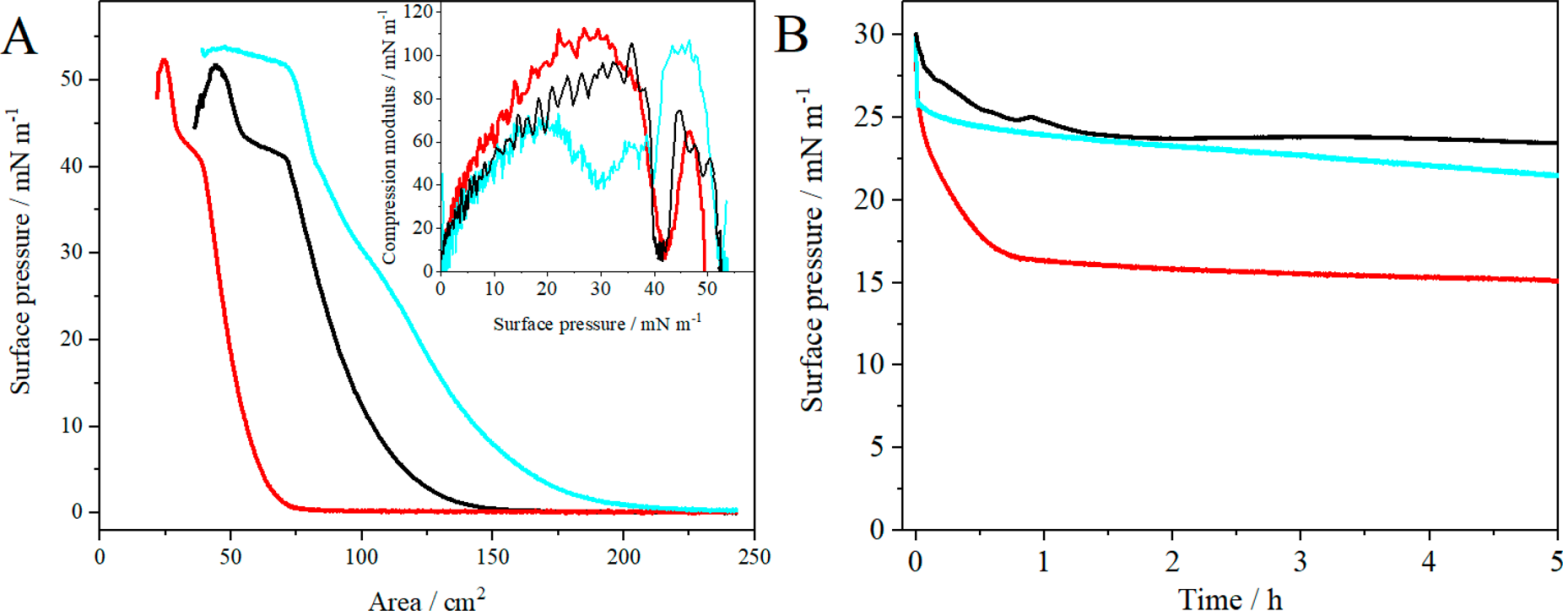
(A) Surface pressure–trough area (π–*A*) isotherms of the 1:1:1 DOPC/Chol/SM monolayer (black) and the layers formed by spreading the liposomes (red) and proteoliposomes with HMG-CoA reductase (0.02 mol %) (blue) formed in the Tris-HCl + NaCl buffer (pH 7.4). Inset: Compression modulus vs surface pressure plot (*T* = 21 ± 1 °C). (B) Surface pressure vs time plots for the 1:1:1 DOPC/Chol/SM monolayer (black) and the layer of liposomes (red) and proteoliposomes with HMG-CoA reductase (0.02 mol %) (blue).

The isotherms of the 1:1:1 DOPC/Chol/SM layers, which were obtained by spreading the ternary lipid mixture solution prepared in chloroform/methanol, liposomes, and proteoliposomes with HMG-CoA reductase (0.02 mol %), are shown in Figure 3. The characteristic parameters of these layers are listed in Table 1. A reorganization of the structure of the 1:1:1 DOPC/Chol/SM (raft) monolayers^35,36^ was observed at high surface pressures (π > 40 mN/m). The partial collapse typical of these types of layers is reflected by a decrease in the compressibility modulus (*C*_s_^–1^) to zero at a surface pressure of 40 mN/m.

**Table 1:** Characteristic Parameters of the Obtained Layers

	π_coll_(mN/m)	*C*_s_^–1^_max_(mN/m)	*C*_s_^–1^_π=30 mN/m_(mN/m)
DOPC/Chol/SM mixed monolayer	53.3 ± 1.8	110 ± 9 _*π=35 mN/m*_	93 ± 7
spread liposomes	49.9 ± 0.5	112 ± 5 _π=27 mN/m_	109 ± 6
spread proteoliposomes with 0.02%HMG-CoA reductase	51.8 ± 2.5	102 ± 3 _π=45 mN/m_	43 ± 3

The shape of the isotherm obtained after spreading the liposomes resembled that of the ternary mixture of lipids (Figure 3). This shows that, upon the spreading of the liposomes, the surface properties of the lipid layer remain the same as those of the ternary mixture. The difference in the position of the two isotherms versus the area of the trough results from differences in the lipid concentration and volume of the two solutions spread at the interface. Nevertheless, the smaller area value corresponding to the spreading of liposomes may indicate that a more compact structure other than a homogeneous monolayer was obtained. Additionally, both layers have similar maximum compressibility modulus values (Table 1), which correspond to the range between the liquid-expanded and liquid-condensed phases, and this demonstrates that the organization of the lipid components at the air–water interface is similar.

The isotherm recorded after spreading the proteoliposomes showed a strong expansion of the layer compared to that obtained for the spreading of the liposomes (Figure 3). The isotherm of the proteoliposomes (blue curve) shifted toward larger areas. The value of the area obtained at approximately 30 mN/m increases by about 50 cm^2^, which is a 100% increase compared to the liposome-derivative isotherm. Since the same volume of both solutions was spread (200 μL), it can be concluded that due to the large size of HMG-CoA reductase^24,61^ the lipid components have to be less tightly packed in the layer. However, the overall expansion of the layer is a result of more fluid lipid film between more compact lipid domains with embedded protein in the lipid layer at the air–water interface. Moreover, the addition of the protein at a concentration of 0.02 mol % (vs lipids) causes a visible inflection in the isotherm at a surface pressure of 30 mN/m, which corresponds to the surface pressure value observed in real cell membranes.^48,49^ This can also be visualized by the minimum in the *C*_s_^–1^ vs π plot (inset in Figure 3), which is not observed in the isotherm recorded after spreading the empty liposomes and may result from the change in the organization of lipids in the presence of protein or from the changes in the HMG-CoA reductase conformation upon compression.^35,36^ Thus, it can be concluded that some lipid–protein interactions occur at the physiological surface pressure of the membranes.

The second inflection observed at the isotherm at approximately 40 mN/m corresponds to the reorganization of the structure observed for both the liposomes (red isotherm) and the ternary lipid layer (black isotherm). Interesting changes were observed in the isotherm shape and in the compression modulus values. Until the surface pressure of 30 mN/m, the layer formed by the proteoliposomes was in the liquid-expanded phase (*C*_s_^–1^ below 70 mN/m); hence, it was less organized and more liquid compared with that obtained by the spreading of liposomes (Table 1). Further compression of the proteoliposome layer resulted in the above-mentioned change in its organization, at approximately 40 mN/m. However, the compression modulus value did not reach zero, as in the case of both the liposome and ternary mixture layers. This suggests that no partial collapse occurred in the proteoliposome layer at this surface pressure. Further compression of the proteoliposome layer resulted in an increase in the compression modulus, which reached a maximum of 102 mN/m at a surface pressure of approximately 45 mN/m. This suggests the slow expulsion of the protein from the layer to generate a more ordered lipid assembly.

The layer formed during the spreading of liposomes at the air–solution interface was significantly less stable (Figure 3B) than the 1:1:1 DOPC/Chol/SM monolayers formed from the mixture of lipids in chloroform/methanol on the PBS buffer subphase^35,36^ or on the Tris-HCl buffer subphase. However, the proteoliposome-derived layers were more stable in time (light blue), and the surface pressure remained constant for many hours, as in the case of the monolayer formed by spreading the sample of the ternary mixture of lipids in the organic solvent (black) (Figure 3B). It seems that the protein penetrating the raft membrane several times forms a scaffold that stabilizes the raft layer structure, similar to ATPase.^13^

**Figure 4:**
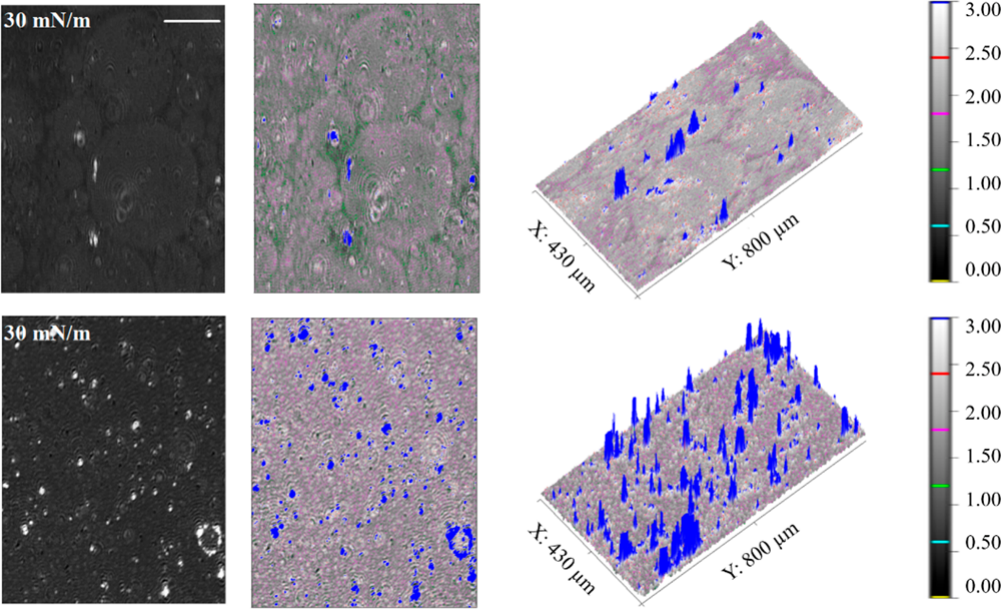
BAM pictures obtained at a surface pressure of 30 mN/m for layers formed by spreading the liposomes (upper panel) and the proteoliposomes with HMG-CoA reductase (0.02 mol %) (lower panel) formed in the Tris-HCl + NaCl buffer (pH 7.4) (*T* = 21 ± 1 °C). The scale bar is 100 μm.

The Brewster angle microscopy images recorded after spreading the liposomes indicate the coexistence of the phases as well as phase separation (Figure 4, upper panel).

Larger oval domains are observed with a tighter packing of molecules with increased layer thickness. It can be concluded that structures are slightly different from those characterizing a monolayer. The formation of local regions with bilayer properties with embedded HMGR enzyme cannot be ruled out. The BAM images of the proteoliposome-derived layer show a more homogeneous structure, with a relatively greater thickness than for the liposome-derived layer (Figure 4). In addition, the presence of larger structures protruding above the surface of the lipid layer, most likely indicative of the presence of the protein in the membrane, can be observed (blue structures in Figure 4, lower panel). They are surrounded by domains of increased thickness, suggesting the local formation of a bilayer also at the air–water interface. This observation was confirmed by the AFM measurements of the layers transferred onto a solid support using the Langmuir–Blodgett method (Figure S3).

### Activity Measurements: UV–Vis Spectrophotometry

**Figure 5:**
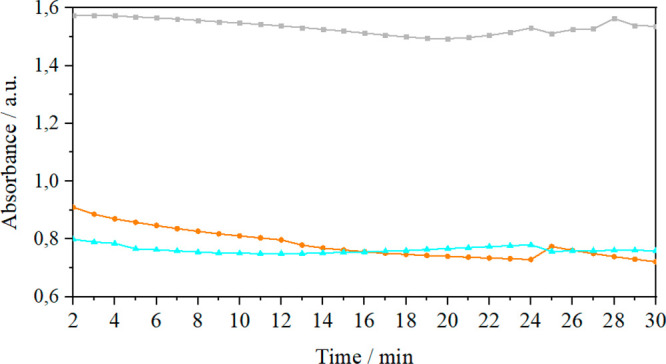
Measurements of the changes in NADPH absorbance over time for the HMG-CoA reductase (catalytic domain) solution (●) and the HMG-COA reductase solution (whole protein) (▲). Absorbance of NADPH in the absence of the catalysts (■). The concentration of the NADPH solution was 4 × 10^–4^ M.

The activity of HMG-CoA reductase in solution and reconstituted in model lipid membranes was determined by measuring the decrease in the NADPH concentration, which was done by measuring the absorbance at 340 nm (Figure 5).

Based on the dependencies presented in Figure 5, it can be concluded that in the absence of HMG-CoA reductase, when there is no catalytic reaction and therefore NADPH is not oxidized to NADP^+^, the maximum absorbance is approximately 1.6 and does not change significantly over time. The presence of only the catalytic domain or the whole reductase in solution leads to the above-mentioned oxidation of NADPH due to its catalytic activity. Therefore, a decrease in the NADPH concentration and, consequently, a decrease in the maximum absorbance value at 340 nm were observed. It should also be noted that during the 30 min of the experiment, the activity of both species (catalytic domain and whole protein) did not change significantly (SD = 3.2 and 2.2% for the catalytic site and the entire enzyme, respectively).

**Figure 6:**
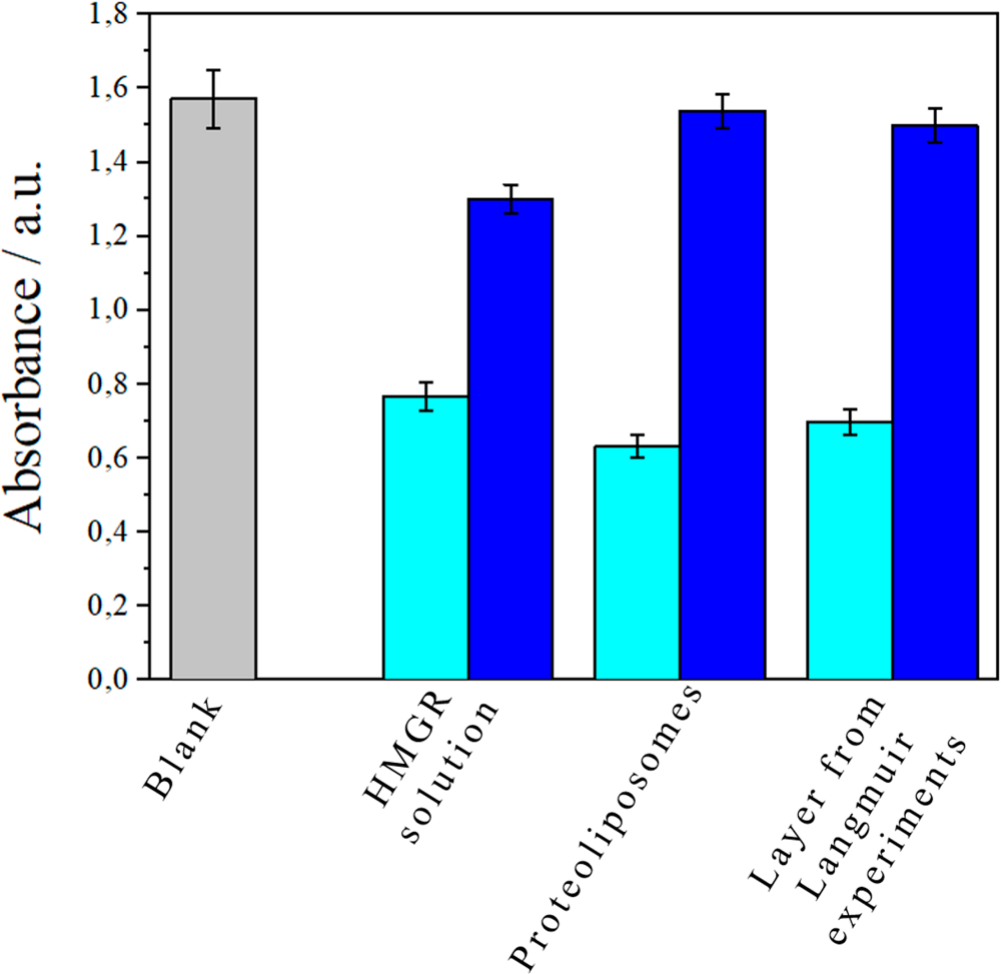
Measurements of the HMG-CoA reductase activity (light blue) and its inhibition by fluvastatin (1 × 10^–5^ M, blue) after 10 min of reaction. Blank: a 4 × 10^–4^ M NADPH solution (gray).

The activity measurements were then repeated for the three types of samples mentioned in the Experimental Methods section. Interestingly, the catalysis of the reaction by the protein in each of the investigated systems was clearly observed from the beginning of the experiment. First, a decrease in the absorbance value of 48% (±1%) in the presence of HMG-CoA reductase was observed (Figure 6). The absorbance of the lipid-layer-bound reductase (proteoliposomes) was even lower than that of HMG-CoA reductase in solution. The reaction occurring when HMG-CoA reductase is exposed at the interface between the lipid membrane and the solution seems to be more effective. Rupture of the vesicles was not observed and was less favorable because there were no ions or detergents in the solution.^51^ The activity of the HMG-CoA reductase incorporated into proteoliposomes and spread at the air–buffer solution interface (Tris-HCl with NaCl) was also determined (Table 2). The proteoliposome incorporation method does not affect the enzyme activity, as clearly shown by the similar absorbance values obtained for the HMG-CoA reductase in solution and incorporated into proteoliposomes. Therefore, the hypothesis that HMG-CoA reductase is an example of a transmembrane protein that can be conveniently studied in a lipid environment and can maintain its activity over time has been confirmed.^1^

**Table 2:** Activity of HMG-CoA Reductase and Its Inhibition in the Presence of Fluvastatin

sample	HMG-CoA reductase activity (units/mg P)	inhibition (%)
HMG-CoA reductase solution	0.80 ± 0.06	82 ± 1
proteoliposomes	0.88 ± 0.03	95 ± 3
layer from Langmuir experiment	0.69 ± 0.05	92 ± 1.5

The function of the enzyme in each system was studied in the presence of an inhibitor, fluvastatin. Inhibition of the catalytic site of HMG-CoA reductase by the statin resulted in the inhibition of reaction 2, which in turn prevented the oxidation of NADPH to NADP^+^ (Figure 5). Interestingly, the absorbance of NADPH in the presence of fluvastatin and protein increased in all three samples analyzed. The high absorbance value shows that this statin inhibits HMG-CoA reductase in reaction 2 when the protein is immobilized in the model membrane systems. It is worth noting that strong inhibition (almost 100% compared to that of the reference sample) is observed for the proteoliposome-bound reductase. This may demonstrate that the reductase catalytic site is exposed to the aquatic environment, resembling real cell conditions in which the catalytic domain is exposed to the cytosol.^24,61^ The fact that the strongest inhibitory effect of fluvastatin was observed for proteoliposomes may also be related to the facile transfer of fluvastatin across the membranes into the vesicle due to its lipophilic properties.^37^ A 92% inhibitory effect was also observed when fluvastatin was added to the subphase, on which the proteoliposomes were spread to form a protein-rich layer. The effective inhibition of HMG-CoA reductase incorporated into the membrane at the interface shows that the HMG-CoA reductase orientation is appropriate for observing both its activity and inhibition.

## Conclusions

In this study, we demonstrate the successful incorporation of HMG-CoA reductase, the enzyme responsible for the synthesis of cholesterol, into liposomes. The lipid mixture used to prepare the liposomes and proteoliposomes containing HMG-CoA reductase was composed of DOPC/Chol/SM in a 1:1:1 molar ratio, which has been shown in our previous studies to be optimal for recreating a lipid system whose composition and surface properties mimic those of the lipid rafts of cell membranes.^35,36^ The slightly larger than usually size of the liposomes (approximately 390 vs 200 nm) was attributed to possible repulsive forces between the different lipid components and the presence of double bonds in the DOPC molecules. The liposomes were characterized by a high degree of curvature, as shown by the fluorescence microscopy results. The significant expansion of the liposome size due to the incorporation of HMG-CoA reductase suggests the effective insertion of the protein into the liposome structure. We demonstrated that the spreading of lipid raft liposomes and proteoliposomes using the Langmuir technique enables the formation of stable thin lipid films at the air–water interface. A similar methodology was previously employed by us to study Na^+^/K^+^-ATPase and allowed us to monitor its behavior without the influence of any solid support, under cell membrane environment conditions.^13^ In the present work, we showed that the activity of HMG-CoA reductase is conserved in the raft model film at the air–water interface, which suggests that such an approach may be successfully used for different types of proteins. We have shown not only that the HMG-CoA reductase protein remains active at the air–water interface but also that its inhibition process can be easily monitored by changing the concentration of the components of the catalytic reaction. Therefore, both lipid raft systems constructed–vesicles and layers at the air–water interface–can be proposed for the investigation of other integral membrane proteins that are exposed to inhibitors/activators considered to be potential drugs. The results of this study together with those of our previous studies showing the influence of statins themselves on the properties of model lipid rafts^35,36^ and the impact of statin lipophilicity on their interactions with model lipid membranes^37,62^ allow us to have much broader insight into the processes taking place in the lipid membranes containing HMG-CoA reductase under the influence of selected drugs.

## Supporting Information Available

Additional fluorescence microscopy images showing the vesicle stability over time; two techniques used to visualize both vesicles (their size and shape) as well as layers resulting from the LB transfer of liposomes and proteoliposomes to a solid support; and atomic force microscopy method showing the thickness of the layers and the platforms that are formed in the presence of the tested HMG-CoA reductase (PDF)
