# Supplementary material for: Model Lipid Raft Membranes for Embedding Integral Membrane Proteins: Reconstitution of HMG-CoA Reductase and Its Inhibition by Statins
Source: Langmuir. 2022 Nov 6;38(45):13888–97. doi: 10.1021/acs.langmuir.2c02115 (PMC9671039; doi:10.1021/acs.langmuir.2c02115)
Supplement: Supplementary file 1 — la2c02115_si_001.pdf [file la2c02115_si_001.pdf]

Supporting Information

Model lipid raft membranes for embedding integral membrane proteins - reconstitution of HMG-CoA reductase and its inhibition by statins

Michalina Zaborowska[1], Dorota Matyszewska[2], and Renata Bilewicz[1]*

[1]*Faculty of Chemistry, University of Warsaw, Pasteura 1, 02093 Warsaw, Poland*

[2]*Faculty of Chemistry, Biological and Chemical Research Centre, University of Warsaw, Żwirki i Wigury 101, 02089 Warsaw, Poland*

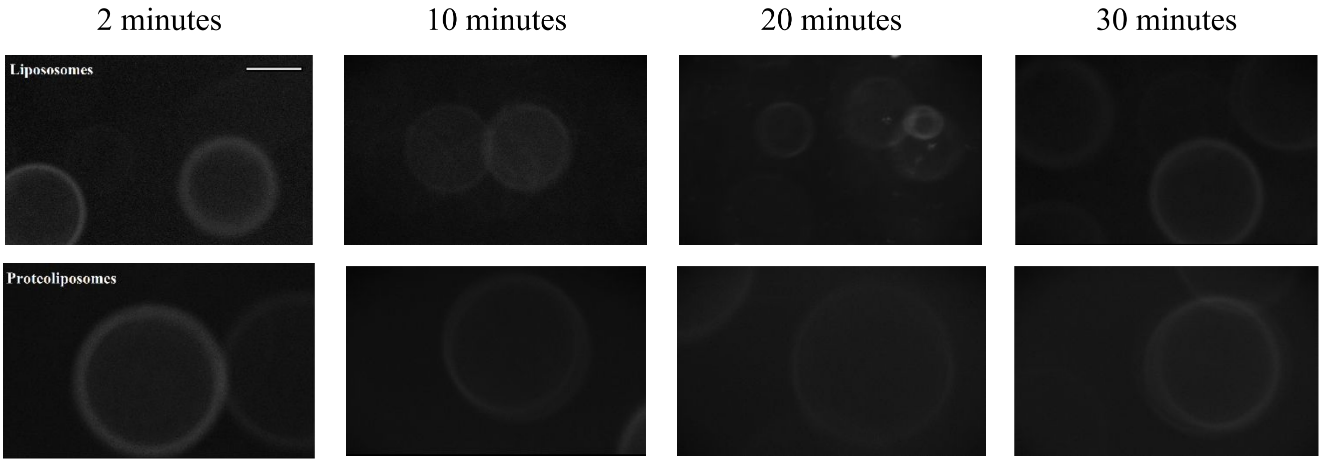

Figure S1 Visualization of the liposomes and proteoliposomes doped with NBD-cholesterol using fluorescence microscopy in time.

Cryogenic Transmission Electron Microscopy (cryo-TEM)

Liposome and proteoliposomes dispersions (3 µl) were plunge-frozen onto glow-discharged Quantifoil R2/1 holey carbon grids with 2nm additional carbon using a Thermo Fisher Vitrobot Mark IV. Prior to plunge freezing a small amount of 10 nm gold beads (Protein A Colloidal Gold, Antibodies-online GmbH Germany) were added to the final solution (concentration of 1:10) to allow for further tomograms reconstruction.

Two-dimensional electron cryomicroscopy images were taken on a Thermo Fisher Glacios TEM operating at 200 kV, equipped with a 4k × 4k Falcon 3EC direct electron detection camera at a magnification of 92 k, which corresponds to a pixel size of 1.587 Å at the specimen level. The defocus was set to 3 µm, and the total electron dose was approximately 50 e/Å^2.

Electron cryotomography was performed at a magnification of 120kx, corresponding to a pixel size of 1.26 Å at the specimen level. Specimens were tilted from approximately −60° to +60° with a 3° increment. The defocus was in a range of 3–6 μm, and the total dose for each tilt series was around 130 e/Å^2.

The cryo-TEM image shows different size of unilamellar vesicles from around 100 nm to 700 nm (Figure S2/A). As can be seen from the above dependences (Figure S2/B), both LUVs and SUVs contain an active protein. However, the fraction of smaller vesicles alone shows less activity, which indicates that more protein in active form is contained in larger vesicular structures.

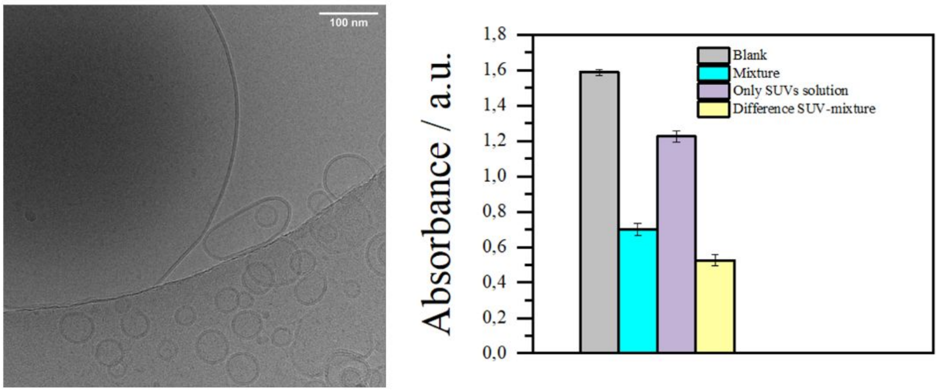

Figure S2 A) Cryo-TEM image visualizing proteoliposome sample; B) HMG-CoA reductase activity measurements depending on the size of vesicles.

Atomic Force Microscopy (AFM)

Prior to the deposition, the mica substrate was cleaned by peeling off the adhesive tape, and then the surface was washed with chloroform. The contact angle measurements showed hydrophilicity of mica plates (~ 35%). The liposome- and proteoliposome-derived layers were transferred by the Langmuir-Blodgett method by the vertical withdrawal of the substrate. For AFM imagining, the Dimension Icon (Bruker, Billerica, MA, USA) in Peak Force QNM mode was used. The z-piezo frequency was modulated at 2 kHz. In order to take the images, ScanAsyst Fluid+ probes with the elasticity constant K = 0.7 N/m were employed.

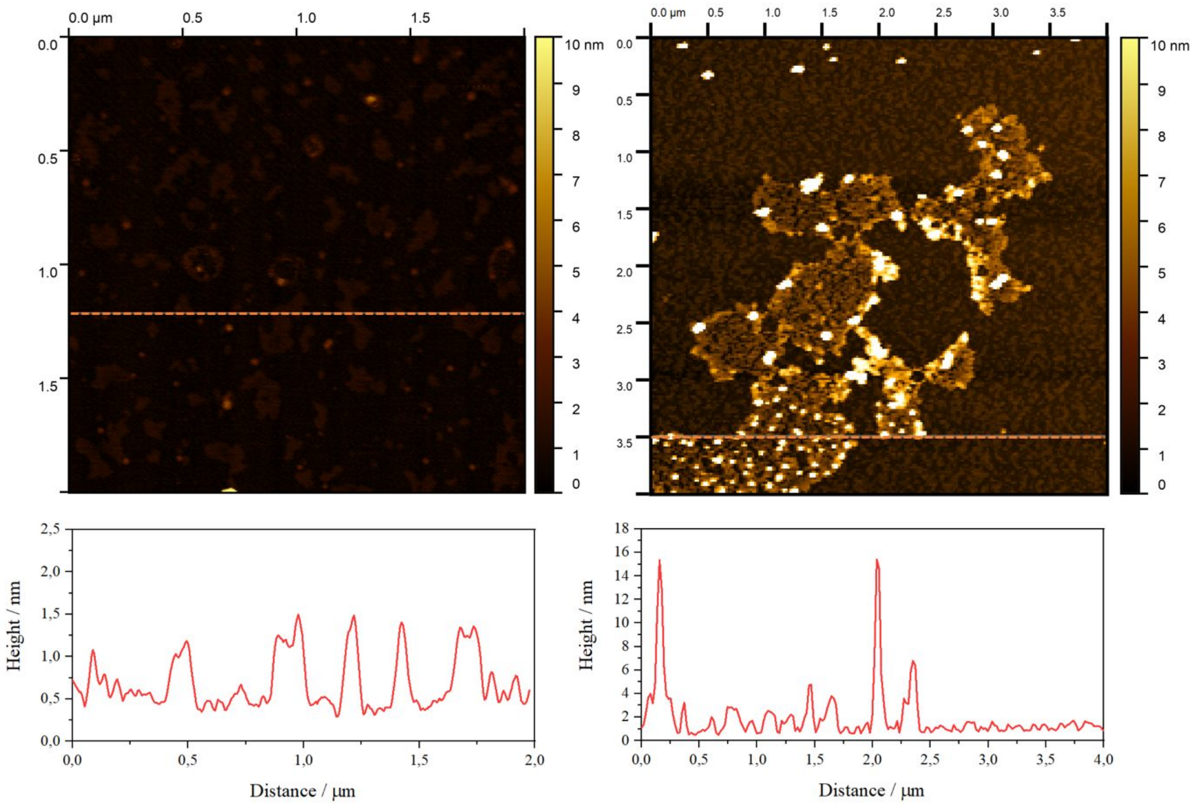

Figure S3 Upper panel: AFM images of liposome (left) and proteoliposomes (right) derived layers; Lower panel: vertical profiles corresponding to yellow dashed lines in the above AFM images.

Figure S3 shows AFM images for the LB transferred layers (liposome- and proteoliposome-derived) from the air-water interface. The results indicate the formation of a monolayer with local thickenings, thus the presence of domains characteristic of lipid rafts. The matrix is composed of a more fluid lipid layer (most likely formed by DOPC) with a thickness of about 0.7 nm. The thickness of the observed more packed platforms is estimated to be about 1 nm. On the other hand, the presence of the protein in the layer causes the formation of the large platforms with a thickness corresponding to a bilayer. The HMGR is located in such platforms and protrudes from the layer by about 10 nm.
